# Resistance characteristics of broad-leaf crop canopy in air-assisted spray field and their effects on droplet deposition

**DOI:** 10.3389/fpls.2022.924749

**Published:** 2022-07-15

**Authors:** Shuo Wu, Jizhan Liu, Junquan Zhen, Xiaojie Lei, Yao Chen

**Affiliations:** Key Laboratory of Modern Agricultural Equipment and Technology, Ministry of Education, Jiangsu University, Zhenjiang, China

**Keywords:** air-assisted spray, crop canopy, resistance characteristics, droplet deposition, pesticide spraying

## Abstract

Air-assisted spray technology is widely applied in high-efficiency pesticide applications. The resistance characteristics of the crop canopy reflect its energy dissipation effect on the assisted airflow, connecting the structure of the crop canopy, assisted airflow velocity, and droplet deposition effect. Using a common broad-leaf crop canopy as the research object, the resistance characteristics of the crop canopy in the air-assisted field were investigated in this study by performing theoretical analysis and wind tunnel tests. Further, the feasibility of using the resistance characteristics of the crop canopy was assessed to evaluate its droplet deposition effect. The results showed that under the conditions of different number of leaf layers and initial leaf azimuth angles, the canopy pressure drop experiences a non-linear increasing trend with increasing assisted airflow velocity and that its regression function conforms to the Darcy–Forchheimer function. Moreover, when the initial azimuth angles of single- and multi-layer leaves were 90°–270°, the change rate of the canopy pressure drop with airflow velocity was 7–9 m/s, and there was a critical wind speed. However, with an increasing number of leaf layers in the crop canopy and changes in the initial leaf azimuth angle, the corresponding changes between the maximum canopy pressure drop and resistance coefficient were non-linear. Thus, it is proposed that the resistance characteristics of multi-layer leaves cannot be quantified as the results of the linear superposition of the resistance characteristics of several single-layer leaves—that is, it should be regarded as a whole research object. Combined with the analysis of the influence of the crop canopy resistance on droplet deposition, it is considered that when the crop canopy has multiple leaf layers in the airflow direction, the existing air-assisted spray technology cannot guarantee droplet deposition and canopy penetration simultaneously.

## Introduction

Crop protection is an important agronomic practice that helps ensure crop yield and quality, with pesticide usage being one of the more effective and widely employed crop-protection methods (Davydov et al., 2018). However, droplet drift, poor canopy penetration, and poor target deposition in pesticide spraying can lead to problems such as pesticide and water wastage, environmental pollution, and food safety concerns (Carvalho, 2006).

Air-assisted spray technology can reduce droplet drift and improve canopy penetration and droplet deposition uniformity by transporting pesticide droplets to the target surface and driving canopy leaves by means of airflow. This method is simple, reliable, and easy to control, making it one of the most widely used spray techniques (Hong et al., 2018). Its integration with pesticide adjuvants, electrostatic spraying, targeted spraying, variable-rate spraying, and other technologies has also become a development trend in crop protection research (Krogh et al., 2003; Stajnko et al., 2012; Patel, 2016; Abbas et al., 2020).

However, Foqué et al. compared the droplet deposition results of vertical sprays with and without air assistance and found that, in some cases, vertical spray deposition was significantly better without air assistance than with it (Foqué et al., 2012). Similarly, our team has been engaged in the research and development of strawberry pesticide spraying technology and equipment for some time. We found that a continuous increase in airflow velocity does not always improve droplet deposition (Wang et al., 2020), because, although the ability of the airflow to change the physical characteristics of the pesticide—such as the droplet size and motion—effectively to improve the canopy penetration and deposition, the motion of crop leaves affected by the assisted airflow force has an equally important effect on droplet deposition. Not all of the crop leaf motion affected by the assisted airflow force in air-assisted spray technology is positive (Derksen et al., 2008).

Therefore, the authors conducted related research on the motion characteristics of strawberry leaves in an air-assisted spray field and their effects on droplet deposition (Wu et al., 2021). Efficient droplet deposition of the crop canopy required that when a leaf moved due to the assisted airflow, contact was ensured between the front and back of the leaves and the droplets, and a reasonable state of motion was achieved to ensure effective deposition. Moreover, the initial position and attitude of crop leaves relative to the assisted airflow affected their state of motion. When the initial azimuth angle of the strawberry leaves was 90°–270°, the airflow more than the critical wind speed drove the leaves to produce a high-frequency, high-amplitude state of vibration that produced a good deposition effect for droplets of small diameters.

Although the initial position and attitude of crop leaves relative to the assisted airflow and speed of the assisted airflow affect droplet deposition, it can be difficult to obtain the initial position and attitude of all leaves in the crop canopy in real time. Moreover, the group effect of crop leaves makes the movement of the group significantly different from that of a single leaf. Consequently, it can be difficult to evaluate the droplet deposition effect of the crop canopy directly through the initial position and attitude of the leaf group relative to the assisted airflow and assisted airflow velocity.

Based on droplet deposition methods—such as the use of water-sensitive paper—to evaluate the crop canopy droplet deposition effect under different air-assisted spray conditions, an efficient deposition mechanism can be achieved by combining high-speed photography with droplet tracing technology, a widely used research method in the field of crop protection (Sánchez-Hermosilla and Medina, 2004; Wang et al., 2008). However, this traditional method can be hampered by expensive equipment, cumbersome processes, and repetition. How to realize the rapid and low-cost evaluation of the effect of droplet deposition on the crop canopy remains a difficult technical problem.

The resistance characteristics of the crop canopy reflect its effects on the airflow energy dissipation at the macro level, which are closely related to the characteristics of the crop canopy—that is, the number of leaves, their initial positions, and the attitude of the leaves relative to the assisted airflow—and assisted airflow velocity (Lhomme, 1991; Fang et al., 2020). In air-assisted spray operations, the airflow and droplets interact with each other, so the resistance characteristics of the crop canopy are closely related to the droplet deposition effect (Liu et al., 2021a). Clearly, the resistance characteristics of the crop canopy can easily form the basis for establishing the relationship among the crop canopy structure, assisted air velocity, and droplet deposition effect. In addition, it is easy to perform rapid measurement at low cost.

Consequently, a broad-leaved crop canopy was considered the research object in this study. Based on the relevant theories and wind tunnel tests, the resistance characteristics of single and multi-layer leaves in the assisted airflow field were studied. The effects of the number of leaf layers, initial position and attitude of the leaves relative to the assisted airflow, and effect of the assisted airflow velocity on the resistance characteristics were analyzed. The feasibility of evaluating the deposition effect of crop canopy droplets based on the resistance characteristics of the crop canopy was assessed. This research provides a theoretical basis for and insight that will facilitate rapid, low-cost research and development of crop protection technology and equipment.

## Theory

### Motion of broad-leaf crop leaves in air-assisted spray field

As leaves are the basic elements of the crop canopy, their motion in the air-assisted spray field constitutes the mathematical basis of relevant theoretical and experimental studies. In a previous study, we proposed a visual descriptive method for leaf motion in an air-assisted spray field, as detailed below (Wu et al., 2021).

**Figure 1:**
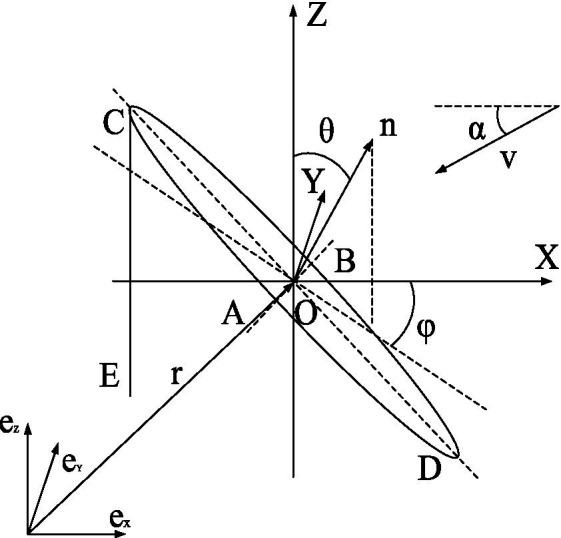
Visual description of leaf movement of broad-leaved crops in air-assisted spray field.

As shown in Figure 1, the base coordinate system, $e_{X}e_{Y}e_{Z},$ is used to represent zero rigid bodies such as the plant roots or ground. The ${\vec{e}}_{X}$ -axis in the base coordinate system, $e_{X}e_{Y}e_{Z},$ is parallel and opposite to the horizontal component of the airflow, $\vec{V}.$ Concurrently, the dynamic relative reference system, OXYZ, for leaf motion can be established in the base coordinate system, $e_{X}e_{Y}e_{Z}.$

The dynamic relative reference system, OXYZ, has the following features: Point O is the mass center of the leaf; the OX-axis is parallel to the horizontal component of the airflow, $\vec{V},$ and has the opposite direction; the OY-axis is vertically orientated; the elliptic ABCD simplifies the representation of the leaf; and line segments CD and AB represent the long and wide axes of the leaf, respectively. Line segment CE represents the slender stem, and point C represents the thin and short petiole connecting the stem and the leaf. The angle between the normal vector, $\vec{n}\;,$ on the front surface of the leaf, ABCD, and the OZ-axis is the inclination angle, θ, of the leaf. The angle between the normal vector, $\vec{n},$ on the front surface of the leaf, ABCD, on the OXY horizontal plane and the OX-axis is the azimuth angle, φ, of the leaf, counterclockwise being the positive direction. The characteristic normal vector, $\vec{n},$ of the position and posture of the leaf relative to the dynamic relative reference system, OXYZ, is (sinθcosφ, −sinθsinφ, cosθ), and the position vector, $\vec{r},$ of the dynamic relative reference system, OXYZ, relative to the base coordinate system, $e_{X}e_{Y}e_{Z},$ is (*x*_o_, *y*_o_, *z*_o_). Therefore, the position and posture of the leaf relative to the base coordinate system, $e_{X}e_{Y}e_{Z},$ can be expressed by (*x*_o_, *y*_o_, *z*_o_, sinθcosφ, sinθsinφ, cosθ), characterized by the vector ${\vec{n}}_{\mathrm{basic}}.$

The motion of the leaf in the base coordinate system in the air-assisted spray field can be expressed as follows:


(1) $${\vec{n}}_{\mathrm{basic},\mathrm{first}}\times A_{\mathrm{trans}}={\vec{n}}_{\mathrm{basic},\mathrm{final}}$$

where ${\vec{n}}_{\mathrm{basic},\mathrm{first}}$ is a vector of the initial position and posture of the leaf relative to the base coordinate system, $e_{X}e_{Y}e_{Z},$ at the beginning; ${\vec{n}}_{\mathrm{basic},\mathrm{first}}$ is a vector of the initial position and posture of the leaf relative to the base coordinate system, $e_{X}e_{Y}e_{Z};$ and $A_{\mathrm{trans}}$ is the position and posture change matrix of the leaf as influenced by the airflow relative to the base coordinate system, $e_{X}e_{Y}e_{Z}.$

### Mechanism of capturing droplets in crop leaves

The process of droplet capture in crop leaves can be complicated, with the droplets, airflow, and leaves interacting during contact. However, the contact between leaves and droplets is the premise of effective deposition. To simplify the analysis, only the influence of the airflow on leaf movement was considered in this study, ignoring the influence of the airflow on the droplets and that of the droplets and leaves on the airflow during the contact process between the leaves and droplets.

**Figure 2:**
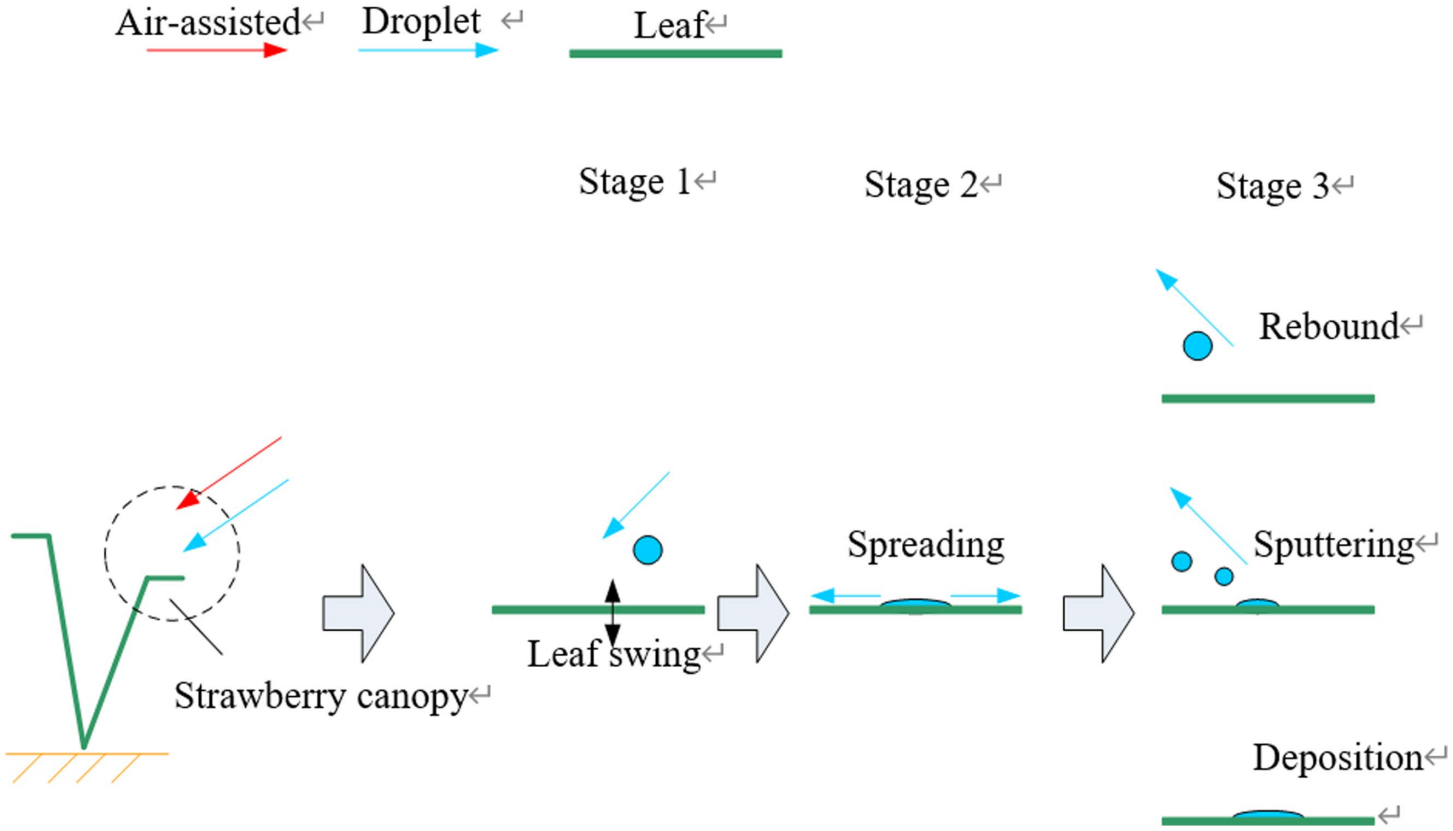
Schematic of droplet capture by leaves.

In a previous study, as shown in Figure 2—combined with the relevant research conclusions of Dorr et al.—the contact process between droplets and plant leaves was thought to occur in three stages: that is, the pre-contact, spreading, and rebound, sputtering, or deposition stages (Dorr et al., 2016; Wu et al., 2021). Droplets are accelerated by the nozzle injection pressure and sprayer airflow in the pre-contact stage, having initial kinetic, potential, and surface energies, with the total energy being E_1_. After a droplet collides with a leaf surface, the initial kinetic energy and potential energy of the droplet are converted into surface energy because of the enlargement of the droplet surface area, with the energy dissipation during the collision being *E*_diss,0–1_. When the diffusion radius reaches its maximum, the droplet begins to shrink under the action of surface tension, during which the energy dissipation is *E*_diss,1–2_.

When the droplet reaches its maximum contraction stage, the total energy, *E*_2_, can be expressed as follows:


(2) $$E_{2}=E_{1}-E_{\mathrm{diss},0-1}-E_{\mathrm{diss},1-2}$$

When *E*_2_ is not sufficiently large to overcome the constraints of the droplet potential energy, adhesion to the leaf, surface tension, and other factors, the droplet does not separate from the leaf, but rather is effectively deposited on its surface. The motion of the leaf influences the initial total energy, *E*_1_, of the droplet as well as the state change of the droplet energy dissipation during its contact with the leaf, thus influencing the effective deposition of droplets on the leaf surface. Efficient droplet capture by the crop canopy requires movement of the leaves, induced by the sprayer airflow, ensuring that both the front and back surfaces of the leaves make contact with the droplets—that is, reasonable motion of the leaves ensures the effective deposition of spray droplets.

**Figure 3:**
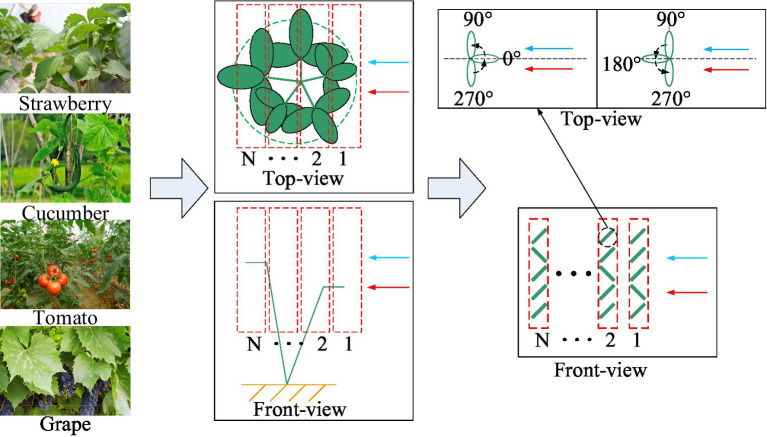
Schematic diagram of quantitative analysis of the droplet capture process in the crop canopy.

As shown in Figure 3, in order to obtain sunlight throughout the day fully, the crop leaves generally grow around. According to the definition of the azimuth angle of the leaf in Section “Motion of broadleaf crop leaves in air-assisted spray field,” the initial azimuth angles of the leaves in the crop canopy relative to the assisted airflow are generally 0°–360°. In this study, the crop canopy was stratified along the direction of the assisted airflow and droplets. The assisted airflow and droplets will attenuate after passing through each leaf layer. In an actual crop canopy, the leaves will overlap, and simultaneously, multiple leaves in a local area range will jointly affect the droplet capture process of the next leaf layer. To analyze the droplet capture process of crop canopy quantitatively, it was assumed for simplicity that the leaves in each layer of the crop canopy would not overlap and each leaf in each layer would only affect the motion and droplet capture process of the corresponding leaves after the direction of their respective airflow and droplets. In a previous study, combining a droplet capture test of crop leaf motion in the air-assisted spray field and the above theoretical analysis, we found that when the initial azimuth angle of the leaf relative to the assisted airflow was 90°–270°, with the appropriate inclination of the applicator fan being the high-frequency and high-amplitude vibration state of the leaf driven by the assisted airflow greater than the critical wind speed, there were good positive and negative uniform deposition effects on droplets of small diameters (Wu et al., 2021). Therefore, on the premise that the initial azimuth angle of the leaf is known, the droplet capture effect can be analyzed. The attenuation analysis of assisted airflow is based on the airflow resistance characteristics of canopy. In this study, the leaf sample layout scheme of the canopy airflow resistance characteristics test and the correlation analysis between the canopy airflow resistance characteristics and droplet capture were based on the above assumptions.

### Description method and theory of crop canopy resistance characteristics

After the assisted airflow passes through the crop canopy, some of its energy is dissipated by it. The resistance characteristics of the crop canopy macroscopically reflect the energy dissipation effect of the crop canopy on assisted airflow, which is closely related to the number of leaves, the initial position and attitude of leaves relative to the assisted airflow, and the velocity of the assisted airflow.

In this study, the Darcy–Forchheimer function in Equation (3) can be used to characterize the resistance characteristics of the crop canopy (Molina-Aiz et al., 2006; Nield and Bejan, 2006; Dullien, 2012):


(3) $$\frac{\partial p}{\partial L}=-\left(D\cdot \mu \cdot v+0.5\cdot C\cdot p\cdot v^{2}\right)$$

To facilitate the wind tunnel test, Equation (3) can be integrated to obtain Equation (4):


(4) $$\Delta p=D\cdot \mu \cdot L\cdot v+0.5\cdot C\cdot \rho \cdot L\cdot v^{2}+A$$

where *p* is the pressure loss of the assisted airflow after passing through the crop canopy ( Pa ), *L* is the length of the crop canopy along the direction of the assisted airflow (*m*), *D* is the viscosity coefficient ( $m^{-2}\cdot s^{-2}$ ), *μ* is the aerodynamic viscosity at the experimental temperature and has a value of $1.79\times 10^{-5}\mathrm{Pa}\cdot s$ , *ρ* is the air density at the experimental temperature and has a value of $1.189\mathrm{kg}\cdot m^{-3}$ , *v* is the assisted airflow velocity ( $m\cdot s^{-1}$ ), *C* is the resistance coefficient, and Δp is the dynamic pressure loss of the assisted airflow through the crop canopy, collectively referred to as the canopy pressure drop.

In this study, the canopy pressure drop and resistance coefficient were used to assess the resistance characteristics of the crop canopy comprehensively.

## Materials and methods

### Leaf sample selection and basic properties

**Figure 4:**
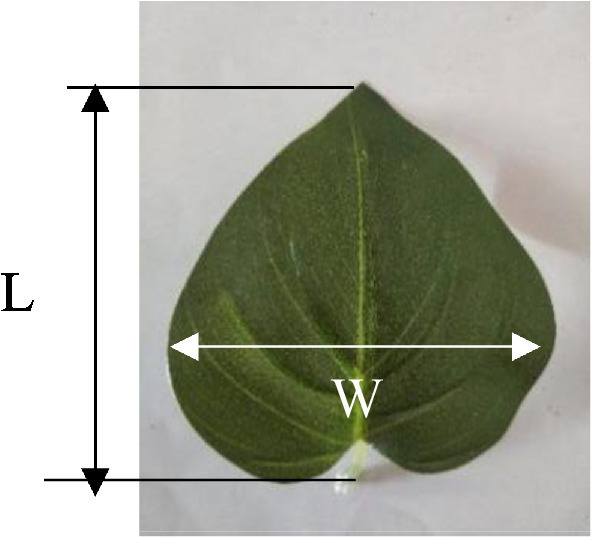
Sample leaf of simulated broad-leaved group.

As shown in Figure 4, simulated broad-leaf crop leaves composed of resin were selected for the experiment, to overcome a series of problems including the individual differences among real crop leaves and the potential effects of repeated tests on their physical properties (Liu et al., 2021a). The sample sizes and physical parameters are listed in Table 1.

**Table 1:** Sample size and physical parameters.

Parameter	Value
Leaf length L (mm)	73.46
Leaf width W (mm)	62.24
Leaf area (mm^2^)	3,078
Density (kg·m^−3^)	900
Petiole length (mm)	15.74
Petiole modulus of elasticity (MPa)	22.5
Elastic modulus of leaf (MPa)	2.36
Ra (μm)	0.16

### Establishment of crop canopy resistance characteristic measurement system

**Figure 5:**
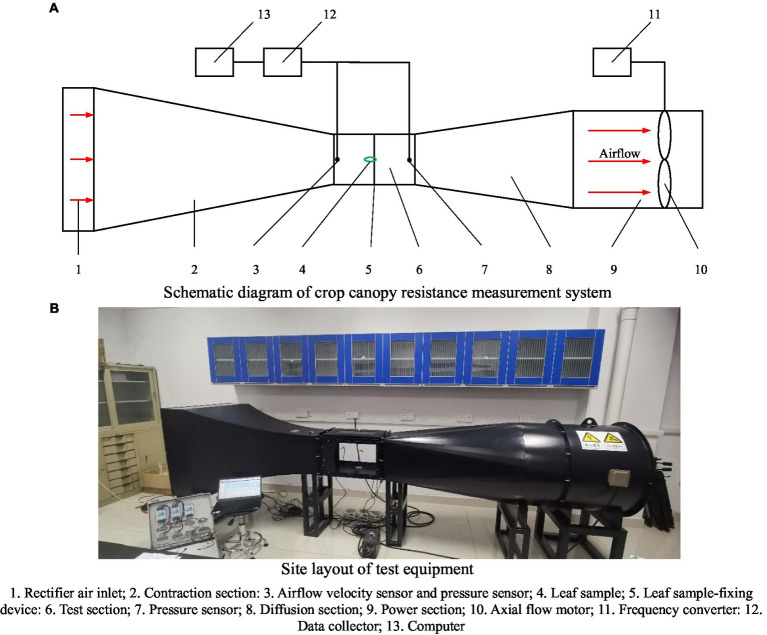
Measurement system of crop canopy resistance characteristics. **(A)** Schematic diagram of crop canopy resistance measurement system. **(B)** Site layout of test equipment. 1. Rectifier air inlet; 2. Contraction section; 3. Airflow velocity sensor and pressure sensor; 4. Leaf sample; 5. Leaf sample-fixing device; 6. Test section; 7. Pressure sensor; 8. Diffusion section; 9. Power section; 10. Axial flow motor; 11. Frequency converter; 12. Data collector; 13. Computer.

As shown in Figure 5, we designed and built a linear wind tunnel measurement system to measure the resistance characteristics of the crop canopy, including the tunnel body, power module, and measurement module (Molina-Aiz et al., 2006).

The tunnel body includes the air inlet, power section, stability section, rectification section, contraction section, test section, expansion section, and air outlet; the power module includes a three-phase DC motor (Shengxiang Machinery Factory, Wuxi, China), frequency converter (Jintian Technology Co., Ltd., Guangdong, China), leaf, and fairing; the measurement module includes a leaf sample-fixing device, hot-wire anemometer (KIMO, Bordeaux, France), and digital micromanometer (DP1000, Hangzhou, China). The sampling frequency of the hot-wire anemometer was 10 Hz, and the accuracy was 0.01 m/s. The range of the digital micromanometer was 0–200 Pa, with an accuracy of 0.1 Pa. The technical parameters of the tunnel body and power module are summarized in Table 2.

**Table 2:** The technical parameters of the tunnel body and power module.

Parameter	Value
Overall size (length × width × height)	4,000 × 900 × 1,350 mm
Motor power (w)	650
Fan impeller diameter (mm)	800
Test section size (length × width × height)	400 × 400 × 600 mm
Wind speed in test section (m·s^−1^)	0.5–20.0
Relative standard deviation of velocity uniformity in test section	≤2.0%
Relative deviation of velocity stability in test section	≤2%
Airflow deflection angle	≤2°

### Airflow resistance characteristic test of multi-position attitude of single leaf

**Figure 6:**
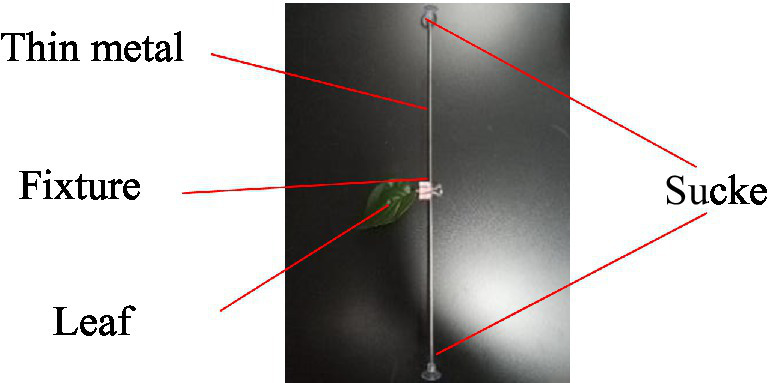
Leaf sample fixation and rotation device.

As depicted in Figures 6, a leaf sample fixing and rotating device was constructed, including a thin metal rod, a clamp, and suction cups. The thin metal rod was fixed in the wind tunnel test section by utilizing suction cups, a clamp was fastened to the rod to affix the leaf sample, and the rod could rotate around the suction cups.

**Figure 7:**
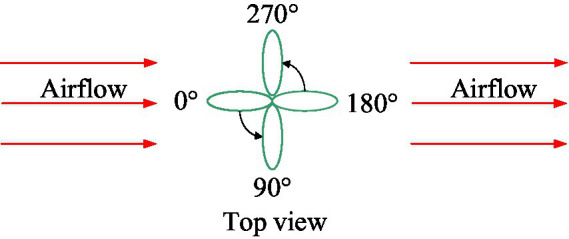
Azimuth adjustment diagram of leaf sample.

The single-factor control variable method was adopted in this study, as illustrated in Figure 7. The initial azimuth, φ, of the leaf sample relative to the assisted airflow was controlled by the rotation of the leaf sample fixing device, and the airflow velocity in the test section was controlled by a frequency converter with a varied range of 0–12 m/s. This airflow velocity range is commonly used in the air-assisted spray. The airflow velocity at the front of the leaf sample was measured using an airflow velocity sensor in front of the sample. The pressures at the front and rear air outlets of the leaf sample were measured using a pressure sensor and micromanometer. The distance between the airflow velocity sensor and the sample was 280 mm. The distance between the pressure sensor and the sample was 280 mm. This information will not be repeated below.

The specific test arrangements are listed in Table 3. To reduce the number of tests, we set eight eigenvalues for the initial azimuth angle of the leaf sample relative to the assisted airflow. Each group of tests was repeated three times, and the average value was calculated.

**Table 3:** Test scheme of airflow resistance characteristics of single leaf at multiple positions.

Test Number	Initial azimuth angle *φ* (°)
1	0
2	45
3	90
4	135
5	180
6	225
7	270
8	315

### Airflow resistance characteristic test of multi-position attitude of multi-layer leaf

**Figure 8:**
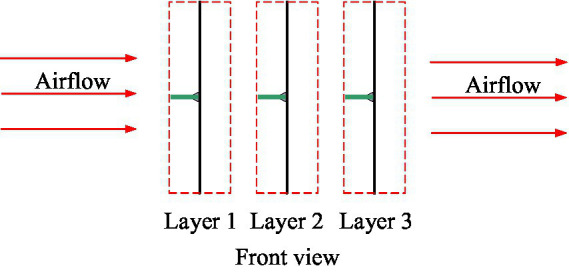
Schematic diagram of leaf sample layer division.

We divided the crop canopy into multiple leaf sample layers in the direction of the assisted airflow, as shown in Figure 8. According to the simplification and assumption mentioned in Section “Mechanism of capturing droplets in crop leaves,” the leaves in the different layers of the crop canopy will not overlap, and each leaf in each layer will only affect the motion and droplet capture process of the corresponding leaves after the direction of their respective airflow and droplets. The influence of each leaf layer on the rear leaf layer can be regarded as the linear superposition of the effects of multiple leaves on the corresponding leaves at the rear. Therefore, we set one leaf sample in each leaf layer. In fact, the number of leaves in each layer of a crop canopy is very large. If the control variable method is used to study the influence of the azimuth difference of the leaves in each layer on the overall resistance characteristics of the crop canopy, the task will become impossible. Therefore, based on the simplified assumption that the resistance characteristics of multiple leaves in each sample layer have linear relationships with those of the individual leaves, we set one leaf sample in each leaf sample layer. The initial azimuth angle, φ, of the leaf sample relative to the assisted airflow was controlled by rotating the leaf sample fixing device, and the airflow velocity in the test section was controlled to 0–12 m/s by a frequency converter. The airflow velocity at the front of the leaf sample was measured using an airflow velocity sensor in front of the sample. The pressures at the front and rear air outlets of the leaf sample were measured using a pressure sensor and micromanometer. To reduce the number of tests, we used two or three leaf sample layers, and the number of leaf samples in each leaf layer was set to two with significantly different initial azimuth angles of 0° and 180°, the specific arrangements of which are listed in Table 4.

**Table 4:** Test scheme of airflow resistance characteristics for multi-position attitude of multi-layer leaves.

Test number	Number of leaf sample layers *N*	Initial azimuth of each leaf layer (*φ*_1_, *φ*_2_, *φ*_3_)
1	2	(0°, 0°)
2	2	(0°, 180°)
3	2	(180°, 180°)
4	2	(180°, 0°)
5	3	(0°, 0°, 0°)
6	3	(0°, 0°, 180°)
7	3	(0°, 180°, 0°)
8	3	(0°, 180°, 180°)
9	3	(180°, 180°, 0°)
10	3	(180°, 180°, 180°)
11	3	(180°, 0°, 0°)
12	3	(180°, 0°, 180°)

Each group of experiments was repeated three times, and the average value was calculated. For convenience, each group of multi-layer leaf tests is described in the form *N*(*φ*_1_, *φ*_2_, *φ*_3_), where *N* is the number of leaf sample layers, *φ*_1_, *φ*_2_, and *φ*_3_ are the initial azimuths of the leaf samples in the first, second, and third layers, respectively.

## Results and discussion

### Airflow resistance characteristics of a single leaf at different initial azimuth angles

In the motion analysis of crop leaves in an airflow field, when the state of motion of the leaves changes suddenly, the airflow velocity corresponding to the change of motion is called the critical wind speed (Shao and Chen, 2011; Tadrist et al., 2015). In this study, the change in canopy pressure drop of leaf samples was an important basis for evaluating whether the state of motion changed.

**Figure 9:**
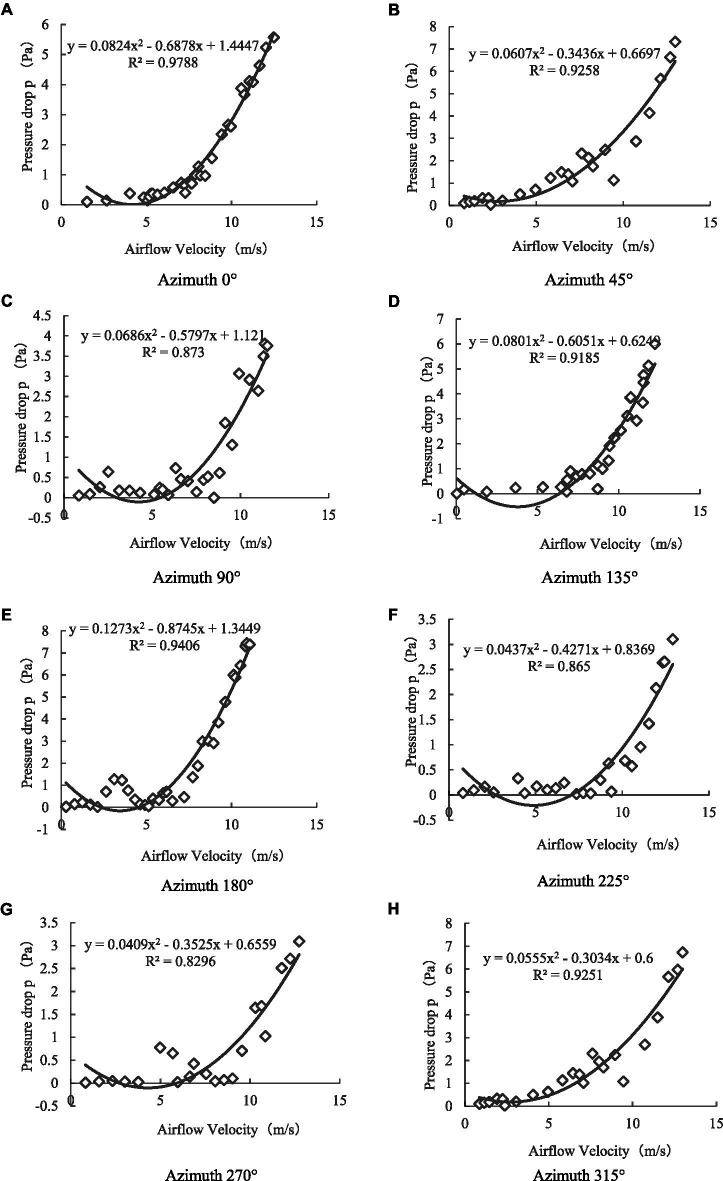
Variation of canopy pressure drop with airflow velocity under different azimuth conditions. **(A)** Azimuth 0°. **(B)** Azimuth 45°. **(C)** Azimuth 90°. **(D)** Azimuth 135°. **(E)** Azimuth 180°. **(F)** Azimuth 225°. **(G)** Azimuth 270°. **(H)** Azimuth 315°.

**Figure 10:**
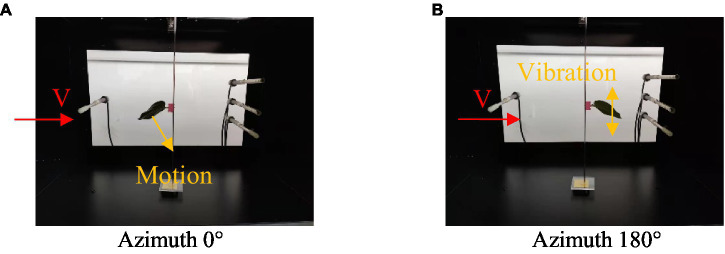
Motion of leaves at different airflow velocities, *V*, and azimuth angles, *φ*. **(A)** Azimuth 0°. **(B)** Azimuth 180°.

Through comparison, as shown in Figure 9, we found that under different initial azimuth conditions, the canopy pressure drop of a single leaf exhibited a non-linear increase with a continuous increase in the assisted airflow velocity. When the initial azimuth angle was 90°, 135°, 180°, 225°, and 270°, the critical wind speed was 7–9 m/s. When the assisted air velocity was less than the critical wind speed, an increase in the assisted air velocity did not significantly improve the leaf canopy pressure drop. However, when the air velocity was greater than the critical wind speed, the leaf canopy pressure drop increased rapidly with increasing assisted air velocity. Moreover, when the initial azimuth was 0°, 45°, and 315°, there was no critical wind speed, marking a sudden change in the canopy pressure drop with increasing assisted airflow velocity. As shown in Figure 10, this finding was obtained because when the initial azimuth of the leaf was 0°, 45°, and 315°, the assisted airflow and front face of the leaf formed an effective airflow load surface, with the airflow load driving the leaf inclination with increasing airflow velocity and the windward area also gradually increasing, resulting in a gradually increasing canopy pressure drop. When the initial azimuth of the leaf was 90°, 135°, 180°, 225°, and 270° and the air velocity was less than the critical wind speed, and the increase in assisted air velocity did not significantly improve the windward area of the leaf. However, when the assisted air velocity was greater than the critical wind speed, the leaf presented an unstable high-frequency and high-amplitude vibration state, greatly dissipating the assisted air energy, resulting in a rapid increase in the canopy pressure drop.

Simultaneously, we performed quadratic polynomial regression fitting on the test data. The obtained curve corresponding to Equation (5) conforms to the configuration of Equation (4), where the determination coefficient *R*>^2^ is 0.83–0.98:


(5) $$\Delta p=K_{1}v+K_{2}v^{2}+A$$

**Figure 11:**
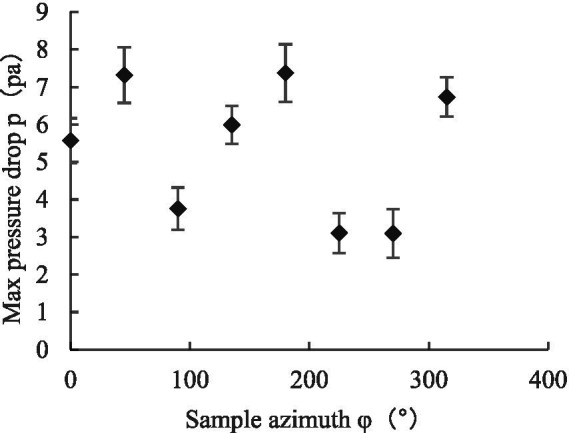
Variation of maximum canopy pressure drop with initial azimuth.

**Figure 12:**
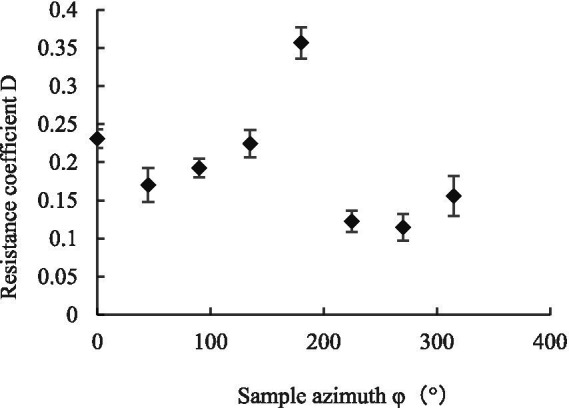
Variation of drag coefficient with initial azimuth.

Referring to Equation (4), we calculated the maximum canopy pressure drop and resistance coefficient C of the leaf under different initial azimuth conditions using the coefficients *K_1_* and *K_2_* of the regression fitting curve of Equation (5), as shown in Figures 11, 12 (Sanz, 2003; Song and Fu, 2020). When the initial azimuth of the leaf was 180°, the maximum canopy pressure drop and airflow resistance coefficient were greater than those at other initial azimuth angles, with values of 7.37 ± 0.77 Pa and 0.35 ± 0.02, respectively. When the leaf azimuth was 90°, 225°, and 270°, the maximum canopy pressure drop and airflow resistance coefficient were less than those at other azimuth positions, with values of 3.75 ± 0.56 Pa and 0.19 ± 0.01, 3.10 ± 0.53 Pa and 0.12 ± 0.01, and 3.09 ± 0.65 Pa and 0.11 ± 0.02, respectively. When the initial azimuth of the leaf was at other positions, the difference between the maximum canopy pressure drop and airflow resistance coefficient was not obvious.

The reason for this finding is that the maximum canopy pressure drop and resistance coefficient of the leaf reflected the dissipation capacity of the leaf to the airflow energy. This energy dissipation capacity includes two parts—that is, when the assisted airflow passes through the leaf canopy, part of the energy is dissipated due to friction, leaf upwind blocking, and other factors and the other part is transformed into the kinetic energy of the leaf. However, when the leaf azimuth changes constantly, the weight of energy dissipation of factors such as friction, leaf upwind resistance, and leaf kinetic energy conversion is an ever-changing process.

### Airflow resistance characteristics of multi-leaf and multi-position attitude

**Figure 13:**
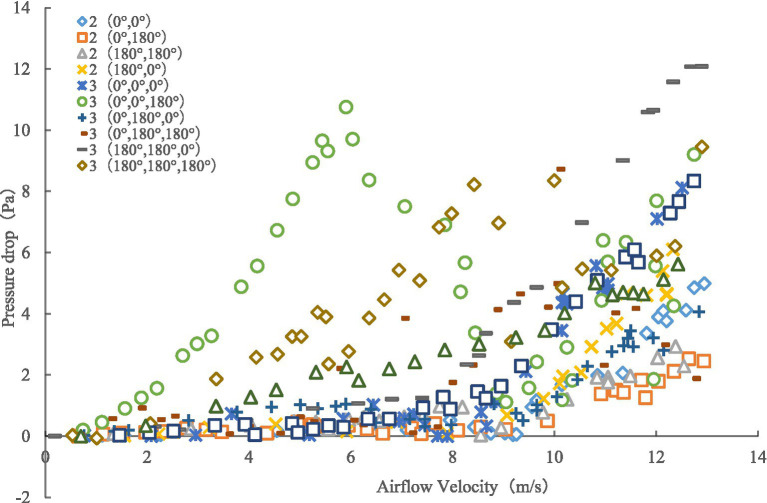
Variation of canopy pressure drop with air velocity with different numbers of leaf sample layers.

Through the comparison in Figure 13, we found that with an increasing number of leaf layers, the canopy pressure drop still increases in a linear nonlinear proportion with continual increases in the assisted airflow velocity, with a critical wind speed in the 7–9 m/s range. When the air velocity is less than the critical wind speed, an increase in the assisted air velocity does not significantly improve the canopy pressure drop. However, when the air velocity is greater than the critical wind speed, the pressure drop increases rapidly.

**Figure 14:**
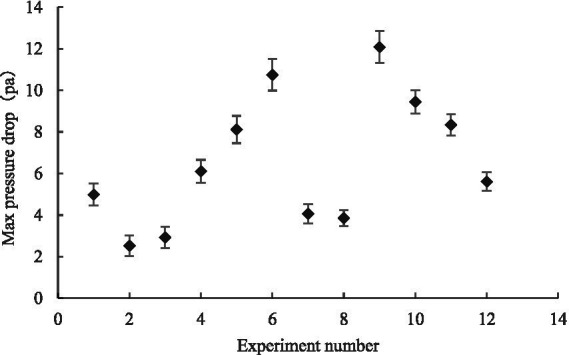
Variation of maximum canopy pressure drop with the number of leaf layers and initial azimuth.

**Figure 15:**
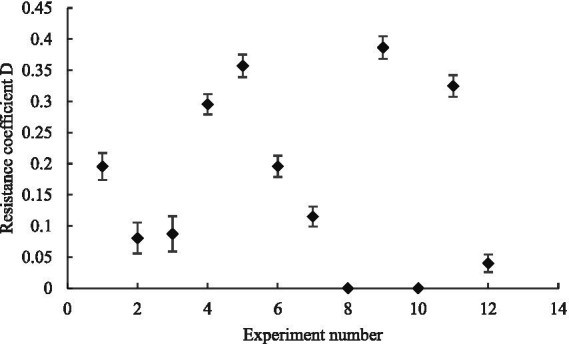
Variation of resistance coefficient with the number of leaf layers and initial azimuth.

We performed quadratic polynomial regression fitting on the experimental data to obtain the curve of Equation (5) and used the coefficients *K_1_* and *K_2_* of the fitting curve of Equation (5) to calculate the maximum canopy pressure drop and resistance coefficient of the leaves under different leaf layers and initial azimuth angles. The comparisons in Figures 14, 15 indicate that when the number of leaf layers is two, the maximum canopy pressure drop and resistance coefficient of the leaf are not more than those of a single leaf and that they have numerical ranges of 2.53–6.10 ± 0.55 Pa and 0.08–0.29 ± 0.55, respectively. When the number of leaf layers is three, the maximum canopy pressure drop and resistance coefficient of the leaf are clearly more than those of the single-layer leaf, and their numerical ranges are 3.83–12.09 ± 0.77 Pa and 0.04–0.38 ± 0.02, respectively. Moreover, the ratio of the number of leaf layers with an initial azimuth of 180° to the number of leaf layers with an initial azimuth of 0° directly affects the maximum pressure drop and resistance coefficient of the crop canopy. When the number of leaf layers with an initial azimuth of 180° is large, the maximum pressure drop of the crop canopy is relatively small. As the number of leaf layers in the crop canopy and the differences in the initial azimuths of the leaves in the layer increase, the corresponding change between the maximum canopy pressure drop and the resistance coefficient is non-linear and difficult to quantify, as the result of the linear superposition of the resistance characteristics of multiple single-layer leaves. Thus, it is recommended that when there are multiple leaf layers in a canopy, it should be regarded as a single research object.

### Relationships between resistance characteristics of crop canopy and droplet deposition effect

In the process of air-assisted spraying, there should be a positive correlation between the energy dissipation effect of the canopy on the assisted airflow and its ability to capture droplets (Cox et al., 2000; Endalew et al., 2010a). Based on this premise, we analyzed the relationship between the resistance characteristics of the crop canopy and the fog droplet deposition effect.

When the crop canopy had only a single leaf in the direction of the assisted airflow, the canopy pressure drop increased non-linearly with increasing assisted airflow velocity, meaning that the droplet-catching ability of the leaf also increased. When the initial azimuth angle of the leaf was 0°–90° or 270°–360°, an increase in the windward load area increased the canopy pressure drop, but the droplet deposition on the back of the leaf could not be guaranteed. When the initial azimuth angle of the leaf was 90°–270°, if the wind speed of the assisted airflow was greater than the critical wind speed, the unsteady high-frequency and high-amplitude vibration state of the leaf increased the canopy pressure drop, with the leaf exhibiting a good droplet deposition effect on both sides, which is consistent with the conclusions of previous studies. Consequently, for single-layer leaves, we could evaluate the corresponding initial azimuth and droplet deposition effect by considering when the canopy pressure drop value changed with the airflow velocity and whether there was a critical wind speed, combining the maximum canopy pressure drop value and resistance coefficient.

When the crop canopy had multiple leaves in the direction of the assisted airflow, the leaves in the first layer inevitably captured most of the droplets when the velocity of the assisted airflow was greater than the critical wind speed, resulting in poor canopy penetration. When the assisted airflow velocity was kept below the critical wind speed and the initial azimuth angle of each layer of leaves was 90°–270°, although canopy penetration could be guaranteed, the droplet deposition effect of the corresponding leaf layer was worse. Moreover, as the number of leaf layers in the crop canopy increased and the initial azimuth angles of the leaves within the canopy changed, the corresponding changes in the maximum pressure drop and resistance coefficient were non-linear and difficult to quantify. It was difficult to judge the number of leaf layers and the specific canopy structure based on the resistance characteristics of the crop canopy with the characteristics of multiple leaves. Consequently, it was considered that existing air-assisted spray technology could not guarantee the droplet deposition effect and canopy penetration at the front layer when the canopy had multiple leaves in the assisted airflow direction.

### Application potential of resistance characteristics in the evaluation of air-assisted spraying effect

Combining CFD technology with field testing is a common method of studying the distribution and attenuation law of the coupled field of airflow and droplets in the inner space of a crop canopy (Endalew et al., 2010b,c). The distribution and attenuation law of the coupling field between the assisted airflow and droplets in the inner space of a complex crop canopy is always a research problem. The existing CFD technology can only simplify the crop canopy into a porous medium model for calculation and analysis. The distribution and attenuation law of the coupled field of the assisted airflow and droplets in the internal space of the crop canopy completely ignores the characteristics of the crop leaves and canopy under airflow stress, and accuracy cannot be guaranteed. Based on the field test of the distribution and attenuation law of the coupling field between the assisted airflow and droplets in the inner space of the crop canopy, the canopy was layered along the assisted airflow direction, and the relationships between the distribution and attenuation and the canopy leaf area index, porosity, resistance coefficient, and other structural characteristic parameters were established (Sun et al., 2015; Sun and Liu, 2019). However, the influence of the motion characteristics of the crop canopy under assisted airflow force on the changes in the canopy leaf area index, porosity, resistance coefficient, and other structural characteristic parameters was still ignored. Liu et al. considered the potential influence of the airflow stress movement characteristics of a crop canopy on droplet deposition (Liu et al., 2021b). However, only under certain working conditions, the canopy deformation characteristics of cotton crop are small, so it is difficult to apply his approach to other crops, and it is impossible to establish a universal and efficient theoretical model. Section “Introduction” mentioned that the evaluation of the droplet deposition effect of the crop canopy under different air-assisted spraying conditions based on water-sensitive paper and other droplet deposition measurement methods has the problems of expensive equipment, a complicated process, and repetition. In this study, the effects of airflow-forced movement characteristics of crop canopy on air-assisted spraying were considered, and the effects of number of the leaf layer, initial position, and attitude of leaves relative to the assisted airflow as well as assisted airflow speed on the resistance characteristics of the crop canopy were analyzed. Finally, the application potential of crop canopy resistance characteristics in air-assisted spraying effect evaluation was verified.

## Conclusion

In general, our experimental data showed that the number of leaf layers and the initial azimuth of leaves in the crop canopy significantly affected the resistance characteristics of the canopy and that these resistance characteristics were also being closely related to the effects of droplet deposition. Using a broad-leaved crop canopy as an example, the following conclusions can be drawn.

Under the conditions of different leaf layers and initial leaf azimuth angles in different leaf layers, the canopy pressure drop increases non-linearly with increasing assisted airflow velocity. The curve equation obtained by regression fitting conformed to the Darcy–Forchheimer equation. When the initial azimuth of the single-layer leaf was 90°–270°, there was a critical wind speed in the 7–9 m/s range, and when the assisted air velocity was less than this critical wind speed, an increase in the assisted air velocity did not significantly improve the canopy pressure drop. However, when the air velocity was greater than the critical wind speed, the canopy pressure drop of the leaf increased rapidly with increasing assisted air velocity.

For a single leaf, when the initial azimuth angle of the leaf was 180°, the maximum canopy pressure drop and airflow resistance coefficient were greater than those of the other initial azimuth positions, at 7.37 Pa and 0.35, respectively. When the leaf azimuth angle was 90°, 225°, and 270°, the maximum canopy pressure drop and airflow resistance coefficient were smaller than those of other azimuth positions, with values of 3.75 and 0.19, 3.10 and 0.12, and 3.09 and 0.11, respectively. When the initial azimuth was in other positions, the differences between the maximum canopy pressure drops and airflow resistance coefficients were not obvious.

When the number of leaf layers was two, the maximum canopy pressure drop and resistance coefficient were not more than those of a single leaf, with values of 2.53–6.10 Pa and 0.08–0.29, respectively. When there were three leaf layers, the maximum canopy pressure drop and resistance coefficient were clearly larger than those of a single leaf, with values of 3.83–12.09 Pa and 0.04–0.38, respectively. Moreover, the ratio of the number of leaf layers with an initial azimuth angle of 180° to the number of leaf layers with an initial azimuth angle of 0° directly affected the maximum pressure drop and resistance coefficient of the crop canopy. The maximum pressure drop of the crop canopy was relatively small when the initial azimuth angle was 180°.

We analyzed the relationships between the resistance characteristics of the crop canopy and droplet deposition effect. For single-layer leaves, we evaluated the corresponding initial azimuth and droplet deposition effect based on whether the canopy pressure drop changed with air velocity and whether there was a critical wind speed and combined the maximum canopy pressure drop and resistance coefficient. When the crop canopy had multiple leaves in the assisted airflow direction, the existing air-assisted spray technology could not guarantee the droplet deposition effect and canopy penetration simultaneously.

Our experiment had several limitations. Although we focused on the resistance characteristics of single and multi-layer leaves in an assisted airflow field, the number of leaf layers in the crop canopy and initial azimuth angle of the leaves in the layer significantly affect their resistance characteristics. However, in the real world, the leaves are different and the leaf population structure of the crop canopy is much more complex than that set in this study. When the assisted airflow passes through the crop canopy, its change law becomes more complex, and the movement law of the leaf population is significantly different from that examined in this study. Moreover, due to connections between stems, many leaves move in concert, which needs to be investigated further. This study indicates that when the number of layers of the crop canopy exceeds 1 in the direction of assisted airflow, air-assisted spraying technology cannot guarantee that every layer of leaves in the crop canopy has a good droplet deposition effect in theory. Solving this problem is of great significance for the research and development of crop protection technologies and equipment.

## Data availability statement

The original contributions presented in the study are included in the article/supplementary material, further inquiries can be directed to the corresponding author.

## Author contributions

SW, JL, and JZ designed the study, performed the experiments and data analysis, and wrote the manuscript. JZ and YC advised on the design of the experiments. All authors contributed to the article and approved the submitted version.

## Funding

The work was supported by the Jiangsu Provincial Key Research and Development Program (BE2020383) and the Changzhou Science and Technology Bureau (CE20202014).

## Conflict of interest

The authors declare that the research was conducted in the absence of any commercial or financial relationships that could be construed as a potential conflict of interest.

## Publisher’s note

All claims expressed in this article are solely those of the authors and do not necessarily represent those of their affiliated organizations, or those of the publisher, the editors and the reviewers. Any product that may be evaluated in this article, or claim that may be made by its manufacturer, is not guaranteed or endorsed by the publisher.
